# The genetic landscape of Lynch syndrome in the Israeli population

**DOI:** 10.1007/s10689-024-00432-w

**Published:** 2024-11-15

**Authors:** Aasem Abu Shtaya, Sofia Naftaly Nathan, Inbal Kedar, Eitan Friedman, Elizabeth Half, Gabi Lidzbarsky, Gili Reznick Levi, Ido Laish, Lior Katz, Lily Bazak, Lilach Peled Peretz, Lina Basel Salmon, Liza Douiev, Marina Lifshitc Kalis, Menachem Schechter, Michal Barzily-Rokni, Nadra Nasser Samra, Naim Abu-Freha, Ofir Hagari-Bechar, Ori Segol, Samar Mattar, Sarit Farage Barhom, Shikma Mordechai, Shiri Shkedi Rafid, Stavit A. Shalev, Tamar Peretz-Yablonski, Zohar Levi, Revital Bruchim, Chana Vinkler, Rinat Bernstein-Molho, Sari Lieberman, Yael Goldberg

**Affiliations:** 1 Recanati Genetics Institute, Faculty of Medicine, Rabin Medical Center – Beilinson Hospital, Tel Aviv University, Petach Tikva, Tel Aviv, Israel; 2 Unit of Gastroenterology, Lady Davis Carmel Medical Center, Haifa, Israel; 3 The Meirav High Risk Clinic, Chaim Sheba Medical Center, Tel-Hashomer, and the Center for Personalized Preventive Medicine, Assuta Medical Center, Tel-Aviv, Israel; 4 Faculty of Medical and Health Sciences, Tel Aviv University, Tel Aviv, Israel; 5 GI Malignancies Prevention Unit, Gastroenterology Department, Rambam Health Care Campus, Haifa, Israel; 6 The Genetics Institute, Rambam Health Care Campus, Haifa, Israel; 7 Department of Gastroenterology, Sheba Medical Center, Tel Aviv, Israel; 8 Department of Gastroenterology, Hadassah Medical Center, Jerusalem, Israel; 9 Medical Genetics Institute, Shaare Zedek Medical Center, Jerusalem, Israel; 10 Genetic Unit, Ziv Medical Center, Tzfat, Israel; 11 The Institute of Gastroenterology and Hepatology, Beer-Sheva, Israel; 12 Faculty of Health Sciences, Ben-Gurion University of the Negev, Beer-Sheva, Israel; 13 Maccabi Health Care Organization, Tel-Aviv, Israel; 14 Institute for Medical Genetics, Wolfson Medical Center, Holon, Israel; 15 Department of Surgery B, Carmel Medical Center, Haifa, Israel; 16 Department of Genetics, Hadassah Hebrew University Medical Center, Jerusalem, Israel; 17 Genetics Institute, Emek Medical Center, Afula, Israel; 18 Rappaport Faculty of Medicine, Technion-Israel Institute of Technology, Haifa, Israel; 19 Sharett Institute of Oncology, Hadassah Hebrew University Hospital, Jerusalem, Israel; 20 Division of Gastroenterology, Rabin Medical Center-Beilinson Hospital, Petach Tikva, Israel; 21 Faculty of Medicine, Hebrew University, Jerusalem, Israel; 22 Susanne Levy Gertner Oncogenetics Unit, The Danek Gertner Institute of Human Genetics Chaim Sheba Medical Center, Tel-Hashomer, Israel

**Keywords:** CMMRD, Disease causing variants, Founder, Lynch syndrome, MMR

## Abstract

Deciphering the spectrum and founder disease-causing variants (DCVs) in specific populations can shape and facilitate the diagnostic process of Lynch Syndrome (LS). The aim of this report was to comprehensively update on the genetic landscape of LS in the ethnically diverse Israeli-Jewish population. The cohort included 1080 carriers from 588 families; some from underrepresented, understudied Israeli ethnic groups recruited from 8 genetic institutes and high-risk clinics throughout the country. Variant classification was performed according to the American College of Medical Genetics criteria. A total of 157 DCVs were identified, 12 are reported here for the first time, and 9 reclassified. *MSH2* DCVs were identified in 286 families (49%). Most DCVs (125/157, 80%) were noted in one or two families only. Sixteen DCVs, each detected in ≥ 5 families, and accounted for LS in 378/588 (64%) families. Constitutional mismatch repair deficiency (CMMRD) was diagnosed in 7 families. Twenty-five carriers (2.3%) had an additional DCV or risk alleles in another cancer susceptibility gene. In conclusion, MMR gene variant distribution in Israel is diverse. *MSH2* is most commonly mutated due to founder DCVs. Though the 16 prevalent LS-associated DCVs were frequently detected in our cohort, none of them is frequently reported in the general population. These data should facilitate variant interpretation, spouse and cascade testing.

**Supplementary Information:**

The online version contains supplementary material available at 10.1007/s10689-024-00432-w.

## Introduction

Lynch syndrome (LS) (OMIM 120435) is the most common hereditary colorectal cancer (CRC) syndrome [1], accounting for 1-3% of all CRCs, with a global incidence of 1:300. LS is inherited in an autosomal dominant manner. It is caused by heterozygous germline disease-causing variants (DCVs) in the DNA mismatch repair (MMR) genes: *MLH1*,*MSH2*,*MSH6*,*PMS2* or by deletions in *EPCAM*. It has recently become clear that defects in each of the LS-associated genes give rise to a distinct entity with a unique pathogenesis, different lifetime cancer risks [2], and mean age at diagnosis [3]. Mutated MMR genes predispose the carrier individual to a substantially increased lifetime risk of a wide spectrum of cancers, mainly CRC and endometrial carcinoma [4–6]. Constitutional mismatch repair deficiency (CMMRD) syndrome (OMIM #276300) is caused by biallelic DCVs in MMR genes, associated mainly with highly aggressive hypermutated pediatric cancer [7].

Tumors are characterized by microsatellite instability (MSI) [8] and/or loss of expression of the relevant protein. MSI cells produce neopeptides that are recognized and targeted by the host immune system [9]. The vast majority of MMRd/MSI-H tumors occur sporadically, due to acquired *MLH1* promoter hypermethylation or somatic DCVs, and are not linked to LS. Thus, for a definitive diagnosis of LS, the identification of a germline DCV affecting one of the MMR genes is required.

Variant classification may pose a diagnostic challenge. Studies have shown that more variants of uncertain significance (VUS) were observed per sequenced gene in individuals who were not from a European White population, in middle-aged adults, and in individuals who underwent testing for disorders with incomplete penetrance [10]. LS has incomplete penetrance and is mostly diagnosed in middle-aged and older adults. Indeed, according to ClinVar, there are 7969 variants in MMR genes classified as DCVs [likely pathogenic (LP) or pathogenic (P)] and over 11,000 variants classified as VUS [11]. To reduce uncertainty, a comprehensive assessment of VUS is needed, based mainly on various subtypes of clinical evidence such as clinical observations and variant segregation data, in addition to pathology in cases of hereditary cancers. Variant classification is especially significant in LS given the need to report incidental findings in MMR genes, which are included in the ACMG secondary findings list [12], and given the latest ASCO recommendation to include the MMR genes whenever germline multigene panel testing is ordered for patients with any cancer, not just LS-associated cancer types [13].

Deciphering the genetic spectrum and variant prevalence in specific populations may shape and facilitate the diagnostic process and guide the testing policy. These include, for example, population screening for *BRCA1/BRCA2* founder DCVs among Ashkenazi or Ethiopian Jews [14, 15] or spouse testing for autosomal recessive conditions associated with high morbidity, such as CMMRD. Currently, population screening in Israel is done only for the founder *BRCA1/BRCA2* DCVs.

A few founder DCVs in MMR genes have been described in Ashkenazi Jews and in some other Jewish ethnic groups, such as Georgian and Ethiopian Jews [16]. Oncogenetic testing for these ethnic groups can be done in a two steps, where common DCVs are tested first, followed by comprehensive sequencing if results are negative. Yet, these recurring DCVs only account for a subset of all cases of LS in Israel, and the full spectrum of DNA-MMR DCVs has yet to be elucidated. In this study we report a comprehensive, multicenter analysis focusing on the genetic landscape of LS in the Jewish population in Israel.

## Patients and methods

### Study population

The study was conducted at the Rabin Medical Center and approved by the Institutional Review Board (Code 0847 approved on 01/2022).

Included were subjects self-identified as Jews who were referred for genetic counseling to 8 different genetic and high-risk clinics between 2004 and 2024. Ethnic distribution was based on patient self-identification. Criteria for genetic testing were based mainly on the revised Bethesda guidelines, cascade testing, and somatic MMRd /MSI-H detection in the course of reflex screening performed since 2020 for CRC and endometrial cancer diagnosed under age 70 years.

Data on ethnic origin, parental consanguinity, and personal and family history of colonic polyps or cancer were collected during clinical genetic counseling. Informed consent for genetic testing was obtained in the clinical setting.

Tumors were tested either by immunohistochemistry (for expression loss) or for MSI – using standard techniques. Germline testing in eligible patients mostly followed a two-stage process: genotyping was performed initially for known founder variants in the MMR genes, and if negative, was followed by next-generation sequencing (NGS) of a multi gene panel testing (MGPT) of cancer susceptibility genes – including the MMR genes. Cascade testing for the familial variant was done in first- or second- degree relatives of carriers.

Analysis of allelic frequency of recurrent DCVs was performed in 15,319 exomes sequenced at Rabin and Hadassah Medical Centers, mostly in cases of intellectual disability, malformations, or prenatal diagnosis.

### Genetic testing

#### Founder DCV testing

Until 2020, founder testing included the 3 Ashkenazi Jewish founder DCVs: c.1906G > C; p.Ala636Pro in *MSH2(NM_000251.3)*, c.3984_3987dup; p.Leu1330Valfs*12 and c.3959_3962del; p.Ala1320GlufsTer6 in *MSH6(* NM_000179.3). Since then, the NanoChip^®^ 400 (Nanogen, Inc.) ONCO PANEL 51 kit (Gamidor Diagnostics Ltd., Israel) was applied, as previously described [16]. Ten MMR variants that have been reported in the Jewish population are included in the kit: *MLH1*: c.1411_1414delAAGA, p.(Lys471AspfsX19); c.1771-1772delGA, p.(Asp591Ter); *MSH2*: c.1906G > C, p.(Ala636Pro); c.970_971delCA, p.(Gln324ValfsX8); c.1277-1G > C; c.1165 C > T, p.(Arg389X). *MSH6*: c.3959_3962delCAAG, p.(Ala1320Glufs); c.3984_3987dupGTCA, p.(Leu330ValfsX12); *PMS2*: c.1970dupA, p.(Asn657LysfsX6), c.2192T > G, p.(Leu731Ter).

#### NGS panel

The different multi-cancer panels used included 13 to 160 cancer genes; all panels included *MLH1*,*MSH2. MSH6*,*PMS2* and *EPCAM*. Most individuals were tested at the Genetics Laboratory of Rabin Medical Center. The remainder were tested in other medical centers or HMOs in Israel, or via and commercially available platforms (Blueprint Comprehensive Hereditary Cancer Panel, Blueprint Genetics, Espoo, Finland; CeGat Panel for Hereditary Tumor Syndromes, CeGaT GmbH, Tübingen, Germany; Color Health Comprehensive Hereditary Cancer Panel, Color Health Inc., Burlingame, CA, USA; Fulgent Genetics Hereditary Cancer Panel, Fulgent Genetics, Temple City, CA, USA; Hadassah Medical Center Cancer Panel, Hadassah Medical Center, Jerusalem, Israel; Invitae Multi-Cancer Panel, Invitae Corp., San Francisco, CA, USA; Maccabi HMO Cancer Panel Maccabi Health Services, Tel Aviv, Israel; and Pronto Diagnostics Multicancer Panel, Pronto Diagnostics Ltd., Tel Aviv, Israel).

#### Variant classification

Variants were classified according to the ClinGen InSiGHT Hereditary Colorectal Cancer/Polyposis Expert Panel Specifications to the ACMG/AMP Variant Interpretation Guidelines for specific genes. Classification is also presented using the point system as suggested by Tavtigian et al. [17].

## Results

Overall, 1080 individuals from 588 unrelated, ethnically diverse families, were diagnosed with LS during the study period. Carriers harbored at least one DCV in the MMR genes. More than half of the carriers (597/1080, 55%) were Ashkenazi Jews, and the others were of non-Ashkenazi/Sephardic origin, from various geographic locations, mostly under-represented in large global databases (Table 1).

**Table 1:** Ethnic distribution of jewish-israeli carriers of Lynch syndrome

Ethnic group	No. of LS patients	No. of LS families (CMMRD families)	% of founder DCVs
Ashkenazi	597	354 (5)	275/354 78%
Afghani, Iranian, Iraqi, Kurdish	137	51 (2)	19/51 37%
Mixed/unknown*	114	81	
Georgian	52	34	32/34 94%
Yemenite	51	15	
North African^†^	48	27	11/27 41%
Egyptian	35	8	
Ethiopian	26	8	7/8 87%
Balkan and Sephardic	16	7	
Bukharin, Caucasus	4	3	
Total	1080	588	

*Ancestors from two or more different ethnic backgrounds

^†^Moroccan and Tunisian

CMMRD, constitutional mismatch repair deficiency; LS, Lynch syndrome

**Fig. 1:**
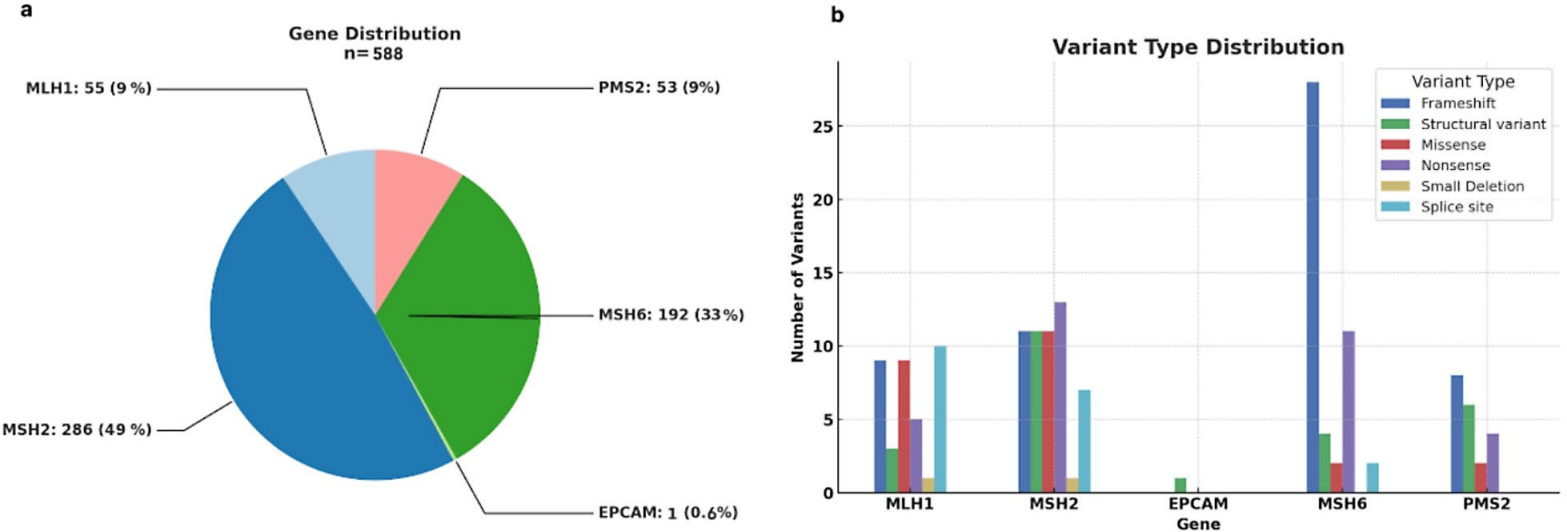
(**a**) Gene distribution among the cohort of 589 families. (**b**) Variant type distribution per gene

The most commonly mutated gene was *MSH2*, noted in 286/588 families (49%). *MSH6* was mutated in 192 families (32.6%), *MLH1* in 55 (9.4%), and *PMS2* in 53 (9%) (Fig. 1a). In total, 157 DCVs were detected: 52 in *MSH2* (33%), 47 in *MSH6* (30%), 38 in *MLH1* (24%), 19 in *PMS2* (12%), and one in *EPCAM* (0.6%). Frameshift variants accounted for 35% of all DCVs, followed by nonsense variants (21%), large (> 1 exon) deletions/duplications (16%), missense variants (15%), splice site variants (12%), and small in-frame deletions (1%). Most DCVs were associated with putative protein truncation. The distribution of the variant types by gene is shown in Fig. 1b.

Of 157 DCVs, 16 (~ 10%) were recurrent, each detected in *≥* 5 families. Collectively, they accounted for genetically diagnosed LS in 378/588 families (64%) (Table 2). Among Ashkenazi Jews, the 3 previously described founder DCVs (MSH2*c.1906G > C, MSH6*c.3984_3987dup, and MSH6*c.3959_3962del) were the most commonly encountered, and detected in 252/354 families (71%). An additional 5 recurrent DCVs accounted for another 9% of cases, making these 8 common variants responsible for 80% of LS cases in Ashkenazi Jews (Table 2; supplementary Table 1).

**Table 2:** The 16 most common variants in this cohort and their carrier frequency according to gnomAD and local population data

	Gene	Variant DNA change; protein change	Number of LS families (*N* = 588)	Subethnic group	gnomAD v.4.1.0 allelic frequency [frequency among AJ]	All of Us allelic frequency	Local DB allelic frequency
1	MSH2	c.1906G > C; p.Ala636Pro	146 (25%)	AJ	0.00074 [0.02702]	0.0016	5/15,014 (0.03)
2	MSH6	c.3984_3987dup; p.Leu1028ValfsTer12	69 (12%)	AJ	0.00037 [0.00676]	0.0008	2/15,014 (0.01)
3	MSH6	c.3959_3962del; p.Ala1320GlufsTer6	37 (6%)	AJ	0.0011 [0.04054]	0.0022	2/15,014 (0.01)
4	MSH2	c.970_971del; p.Gln324ValfsTer8	32 (5%)	Georgian	0.000062	0	0/180
5	PMS2	c.943 C > T; p.Arg315Ter	15 (3%)	Pan-Ethnic *	0.0013	0.0026	8/15,014 (0.05)*
6	PMS2	c.2192T > G; p.Leu731*	14 (3%)	Iranian, Iraqi, Kurdish	0.000062	0	7/4136 (0.17)
7	MSH6	c.3312dup; pGly1105Trpfs*3	11 (2%)	North African	0.00012	0	0/5764
8	MSH2	c.1165 C > T; p.Arg389*	8 (1%)	AJ & North African	0.0000037	0.0004	0/20,778
9	MSH2	del exon 9–10	7 (1%)	Ethiopian	0.00079	NA	0/274
10	MLH1	c.1411_1414del; p.Lys471Aspfs*19	7 (1%)	AJ	0	0	0/15,014
11	MSH2	c.1147 C > T; p.Arg383*	6 (1%)	AJ	0.00012	0	0/15,014
12	MSH6	c.1108_1109del; p.Leu370fs	6 (1%)	Pan-Ethnic	0.000062	0.0002	0/30,638
13	MSH2	c.1861 C > T; p.Arg621*	5 (1%)	Libyan	0	0.0002	1/814(0.12)
14	MSH2	c.2038 C > T; p.Arg680*	5 (1%)	AJ	0.00012	0.0006	0/15,014
15	MSH6	c.1933G > T; p.Glu645*	5 (1%)	Iranian	0.00012	0	0/4136
16	MSH6	c.3743_3744insT; p.Try1249fs	5 (1%)	AJ	0.00012	0.000002	0/15,014

Data are expressed as n (%)

Transcripts are: *MSH*2 (NM_000251.3), *MSH6* (NM_000179.3), *PMS2* (NM_000535.7), *MLH1* (NM_000249.4)

*Variant was seen in multiethnicities in patient cohort but in AJ only in local exomes

AJ, Ashkenazi Jews/ish; DB, database; LS, Lynch syndrome; ND, not determined

The allelic frequency of the 16 recurrent founder DCVs was then estimated in the general ethnically matched local population (Table 2). The *PMS2(*NM_000535.7) c.2192T > G; p.Leu731* DCV was found in 7 of 4136 alleles (0.17%) of individuals who self-identified as being of Iraqi-Iranian-Kurdish Jewish ancestry. Among AJ, 4 DCVs were detected in the exome database, in 17/15,014 (0.11%) AJ alleles. The *PMS2* c.943 C > T; p.Arg315Ter DCV appeared in AJ only in the local variant database but, interestingly, in the cohort, in patients of multiple origins (Table 2).

Recurring variants were also detected in Jews of non-Ashkenazi descent. Twenty DCVs were detected in Iranian and Iraqi Jews, including two, each in > 5 families. Notably, the founder variant *PMS2*:c.2192T > G was detected in 14 families; 56% of Iranian Jews and 24% of Iraqi Jews with LS, who are part of the cohort. Among Georgian Jews, the high-penetrant variant *MSH2*:c.970_971del was detected in 32/34 families (94%), and among Ethiopian Jews, deletion of exons 9 and 10 in *MSH2* was detected in 7 of 8 families (87%). Among Tunisian and Moroccan Jews, the *MSH6* c.3312dup variant was detected in 41% of families (Table 2).

CMMRD, due to bi-allelic DCVs, was diagnosed in 7 seemingly unrelated families: 5 Ashkenazi Jewish and 2 Iranian Jewish. In most of them, the parents were not consanguineous. All cases of CMMRD were due to founder variants: in *MSH2* in one family and *MSH6* in 4 families; the founder variant *PMS2*:c.2192T > G was the cause of CMMRD in both Iranian families with CMMRD. Only one of the patients (previously reported) was a compound heterozygote for a founder variant along with a private variant, c.1444 C > G; p.Arg482Gly in *MSH6*, classified as LP (Table 3) [18].

**Table 3:** Novel MMR DCVs not previously reported in the literature or in insight database

	Gene	Variant	No. of families	ACMG criteria	Point-based classification
1	*MSH2*	c.857_872del; p.Phe286*	2	PVS1,PM2_support, PP4	10 = P
2	*MSH2*	c.1046dup; p.Leu350fs	1	PVS1,PM2_support	9 = LP
3	*MLH1*	c.1528del; p.Gln510fs	1	PVS1,PM2_support	9 = LP
4	*MLH1*	c.92_105del; p.Ala31fs	2	PVS1,PM2_support	9 = LP
5	*MSH6*	c.321_322del; p.Cys108fs	3	PVS1,PM2_support, PP4	10 = P
6	*MSH6*	c.2174del; p.lle725fs	1	PVS1,PM2_support, PP4	10 = P
7	*MSH6*	c.2252del; p.Asn751fs	1	PVS1,PM2_support	9 = LP
8	*MSH6*	c.2611del; p.Ile871fs	1	PVS1,PM2_support	9 = LP
9	*MSH6*	c.3038del; p.Lys1013fs	1	PVS1,PM2_support, PP4	10 = P
10	*MSH6*	c.4002-25_4002-2del	2	PVS1_strong, PM2_support, PP1_supp	6 = LP
11	*PMS2*	c.2148dup; p.Val717fs	1	PVS1,PM2_support	9 = LP
12	*PMS2*	c.2159_2171dup; p.Ile724fs	1	PVS1,PM2_support	9 = LP

ACMG, American College of Medical Genetics and Genomics; DCV, disease-causing variant; LP, likely pathogenic; MMR, mismatch repair [gene]

Transcripts are: *MSH2* (NM_000251.3), *MLH1* (NM_000249.4), *MSH6* (NM_000179.3), *PMS2* (NM_000535.7)

We report 12 novel LP variants that have not yet been described in the literature (Table 4). At the time of diagnosis, none had a ClinVar ID. All 12 were detected in families with significant LS-associated morbidity.

**Table 4:** DCVs reclassified as likely pathogenic from VUS

	Gene	Variant	No. of families	ClinVar	REVEL	ACMG criteria	Point-based classification
				(review status*)			
1	*MSH2*	c.1805T > C p.Leu602Pro	1	3 VUS 2 LP (1 Star)	0.95	PS3, PM2_support, PP3_moderate, PP4	8 = LP
2	*MSH2*	c.797 C > T p.Ala266Val	2	2VUS 1 LP (2 stars)	0.92	PP4_strong, PM2_support, PP3_moderate	7 = LP
3	*MSH2*	c.648_650del p.Ile217del	1	3 VUS	NA	PM4_support, PM2_support, PP3, PP4_moderate, PS3_support^$^	6 = LP
4	*MLH1*	c.1999del p.Asp667fs	1	1 VUS 1 P (1 star)	NA	PVS1, PM2_support	= 9 LP
5	*MLH1*	c.588 + 5G > C	1	2 VUS 1 LP 2 P (1 star)	NA	PVS1(RNA), PS1, PM2_support, PP1, PP4_strong	18 = P
6	*MSH6*	c.3996_3999dup p.Arg1334fs	1	1 VUS 4 LP 2 P (1 star)	NA	PVS1, PM2_support	= 9LP
7	*MLH1*	c.1937 A > G p.Tyr646Cys	3	10 VUS 1 P (1 stars)	0.96	PS4_moderate, PM2_support, PM1, PP3_moderate	7 = LP
8	*PMS2*	c.2186_2187del p.Leu729fs	1	1 B 3 VUS 3 LP 3 P (3 stars)	NA	PVS1, PS4 PM2_support	= 13P
9	*MSH6*	c.1444 C > G p.Arg482Gly	1	2 VUS 1 LP (1 star)	0.8	PM2_support, PM3, PP3_moderate, PP4	6 = LP

^$^(personal communication)

*Stars represent the aggregate review status, or the level of review supporting the aggregate germline classification for a specific record. This value is calculated by NCBI based on data from submitters

B, benign; LP, likely pathogenic; P, pathogenic; VUS, variant of uncertain significance;

Transcripts are: *MSH2* (NM_000251.3), *MLH1* (NM_000249.4), *MSH6* (NM_000179.3), *PMS2* (NM_000535.7)

Nine variants in the cohort were reclassified from VUS to LP/P based on the ACMG criteria. Reclassification was also possible thanks to clinical, pathology, and segregation information. The variants are listed in Table 3 with their corresponding ClinVar classification and review status, along with the point-based classification of our in-house bioinformatics team.

Of the total LS carriers, 25 (2.3%) harbored an additional DCV in another cancer-predisposition gene. In 12 of them (48%), the second variant was a low-penetrant risk allele in *APC* and *CHEK2* (Table 5). The c.5946del; p.Ser1982fs (AKA 6174delT) variant in the *BRCA2*) NM_000059.4 (gene was found in 10/25 double carriers.

**Table 5:** Additional DCVs in other cancer genes among LS carriers

Gene	Variant	No. of carriers
*APC*	c.3920T > A;p. lle1307Lys#	4
*BRCA1*	c.185del; p. Glu23fs	1
*BRCA2*	c.6174del; p. Ser1982fs	10
*CHEK2*	c.470T > C;p. Ile157Thr#	1
*CHEK2*	c.1283 C > T;p. Ser428Phe#	3
*CHEK2*	c.1427 C > T;p. Thr476Met	4
*NBN*	c.1903 A > T;p. Lys635*	1
*POLE*	c.830 A > G;p. Glu277Gly	1

DCV, disease-causing variant; LS, Lynch syndrome

# Currently regarded as risk alleles

Transcripts are: *APC* (NM_000038.6), *BRCA1* (NM_007294.4), *BRCA2* (NM_000059.4), *CHEK2* (NM_007194.4), *NBN* (NM_002485.5), *POLE* (NM_006231.4)

## Discussion

In this multicenter LS cohort, several Jewish ethnic groups displayed recurring, presumably founder, DCVs in the known DNA MMR genes. Notably, DCVs in *MSH2* accounted for about 50% of all Israeli Jewish cases of LS. Seven common recurring DCVs in *MSH2* were detected in both AJ and non-AJ families, contributing to LS in 209/588 families.

These findings are similar to those reported in other genetically homogeneous populations. In Iceland, 2 recurrent DCVs, one in *PMS2* and one in *MSH6*, contributed to LS in 78% of the population [19]. In Finland, one founder DCV in *MLH1* was found to be responsible for 50% of Finnish carriers [20].

Only 19 DCVs were identified in *PMS2* of which 2 were recurrent. This finding may reflect the low penetrance of monoallelic *PMS2*, as the cohort described here consisted mostly of cancer patients diagnosed with LS and family members.

A considerable number of unique DCVs (125/157), found in one or two families, accounted for LS across various ethnicities. These values raise concerns regarding the efficacy of the two-step testing process in which known founder DCVs are tested first, and comprehensive testing is only funded by the ministry of Health for highly suspicious patients. Rather, we advocate for comprehensive genetic testing to ensure rare variants are not overlooked. Rates of detection of recurring DCVs were ethnicity-dependent (Table 1). Although these rates may support the more cost-effective two-step genotyping process, several disadvantages need to be considered: the turn-around time for final results is longer, rare variants may be overlooked, especially in the lower-penetrant *MSH6 and PMS2* genes, and cases with more than one DCV may be missed.

The frequency of DCVs in most minority groups is not well-documented because of their underrepresentation in global databases like gnomAD (https://gnomad.broadinstitute.org) and All of Us (https://www.researchallofus.org). However, an analysis of over 30,000 alleles from the local population has provided an estimate of founder DCVs within various ethnically matched groups. The *MSH2* c.1906G > C variant, the most prevalent founder variant among AJ with LS, has a carrier frequency of 0.03% in AJ both globally in gnomAD v4 and in our local exome database. The combined frequency of the four founder DCVs in AJ detected in our exome data is approximately 1 in 900. The *PMS2* c.2192T > G; p.Leu731* variant, which is considered rare worldwide, was relatively common in our cohort, with a frequency of 1:588 among individuals of Irani-Iraqi-Kurdish descent. Indeed, this variant was associated with CMMRD in the 2 affected seemingly unrelated Iranian families. We did not find evidence of a high frequency of other founder DCVs in other ethnically matched general populations. These findings should guide any regulatory decisions pertaining to population screening and spouse-testing, when applicable.

This work adds information on 21 DCVs, including 12 that have not been previously mentioned in the scientific literature and 9 that were previously classified as VUS (Tables 4 and 3). Our variant classification was based on the integration of clinical data, pathology, molecular pathology, the Insight database [16, 21], and data obtained from our internal datasets regarding the local frequency and distribution of variants. Some of the reclassifications were a result of cascade testing which enabled segregation within families.

It has been reported that VUS are most commonly reported variants among ethnic groups, such as Asian, Black, and Hispanic individuals and Sephardic Jews [10], adding to the health disparities already affecting these populations. The fact that almost half (45%) of our cohort were Sephardic Jews enabled proper diagnosis in this group and the addition of these variants to the global database. Expanding knowledge on DCVs in different subpopulations may also increase awareness among physicians and contribute to a higher rate of referrals for genetic counseling and testing.

Twenty-five genotyped individuals were double heterozygotes, carrying a DCV in a LS gene and an additional DCV in another cancer-susceptibility gene. Of them, twelve individuals had DCVs that are considered low penetrant, and 10 also had a *BRCA2* DCV. The increased use of multigene panels has led to the identification of individuals harboring more than one pathogenic variant in cancer-susceptibility genes. In 2016, Whitworth and colleagues [22] published examples of patients who had, what they termed, multilocus inherited neoplasia alleles syndrome (MINAS). We previously reported a case series of double carriers of LS and hereditary breast and ovarian cancer pathogenic variants, suggesting that double *MSH2/MSH6* and *BRCA1/BRCA2* carriage is not associated with early disease onset or a more severe phenotype [23]. Conversely, we reported a child with “POLE-Lynch syndrome” carrying a de novo *PMS2* DCV and inherited *POLE* DCV, manifested by an aggressive medulloblastoma with a unique genomic signature [24]. More data are needed to further our understanding of the clinical implications and consequences of double heterozygosity so that more targeted surveillance schemes can be offered.

This publication has several limitations. Data were collected from eight genetic institutes and high-risk clinics; however it is not population-based. Thus, in the absence of a national registry, it is challenging to determine the population frequency or the rate of LS in the general population, or among CRC and endometrial cancer patients. Carriers were identified via heterogenous venues, such as reflex screening, founder vriant testing, NGS based testing, cascade screening, or due to incidental findings. In addition, the lack of ongoing clinical update about carriers and family members, limits our ability to provide information on variant penetrance and expression. However, this cohort represents high-risk clinics from all over the country.

## Conclusion

In conclusion, this comprehensive study of LS in the Israeli Jewish population reveals a diverse genetic landscape with several novel and reclassified variants identified. Approximately 10% are recurrent DCVs, mostly affecting the *MSH2* gene. However, these recurring DCVs have a low prevalence in the general population. The data presented here will assist in correct variant classification and will help to improve diagnostic LS accuracy. This is especially important in prophylactic settings, where variant classification is more challenging due to the absence of phenotypic data and the inability to use phenotype-related variant classification criteria.

## Electronic supplementary material

Below is the link to the electronic supplementary material.

Supplementary Material 1

## Data Availability

No datasets were generated or analysed during the current study.
