# Correction: The genetic landscape of Lynch syndrome in the Israeli population

**DOI:** 10.1007/s10689-025-00454-y

**Published:** 2025-03-21

**Authors:** Aasem Abu Shtaya, Sofia Naftaly Nathan, Inbal Kedar, Eitan Friedman, Elizabeth Half, Gabi Lidzbarsky, Gili Reznick Levi, Ido Laish, Lior Katz, Lily Bazak, Lilach Peled Peretz, Lina Basel Salmon, Liza Douiev, Marina Lifshitc Kalis, Menachem Schechter, Michal Barzily-Rokni, Nadra Nasser Samra, Naim Abu-Freha, Ofir Hagari-Bechar, Ori Segol, Samar Mattar, Sarit Farage Barhom, Shikma Mordechai, Shiri Shkedi Rafid, Stavit A. Shalev, Tamar Peretz-Yablonski, Zohar Levi, Revital Bruchim, Chana Vinkler, Rinat Bernstein-Molho, Sari Lieberman, Yael Goldberg

**Affiliations:** 1 Recanati Genetics Institute, Faculty of Medicine, Rabin Medical Center– Beilinson Hospital, Tel Aviv University, Petach Tikva, Tel Aviv, Israel; 2 Unit of Gastroenterology, Lady Davis Carmel Medical Center, Haifa, Israel; 3 The Meirav High Risk Clinic, Chaim Sheba Medical Center, Tel-Hashomer, and the Center for Personalized Preventive Medicine, Assuta Medical Center, Tel-Aviv, Israel; 4 Faculty of Medical and Health Sciences, Tel Aviv University, Tel Aviv, Israel; 5 GI Malignancies Prevention Unit, Gastroenterology Department, Rambam Health Care Campus, Haifa, Israel; 6 The Genetics Institute, Rambam Health Care Campus, Haifa, Israel; 7 Department of Gastroenterology, Sheba Medical Center, Tel Aviv, Israel; 8 Department of Gastroenterology, Hadassah Medical Center, Jerusalem, Israel; 9 Medical Genetics Institute, Shaare Zedek Medical Center, Jerusalem, Israel; 10 Genetic Unit, Ziv Medical Center, Tzfat, Israel; 11 The Institute of Gastroenterology and Hepatology, Beer-Sheva, Israel; 12 Faculty of Health Sciences, Ben-Gurion University of the Negev, Beer-Sheva, Israel; 13 Maccabi Health Care Organization, Tel-Aviv, Israel; 14 Institute for Medical Genetics, Wolfson Medical Center, Holon, Israel; 15 Department of Surgery B, Carmel Medical Center, Haifa, Israel; 16 Department of Genetics, Hadassah Hebrew University Medical Center, Jerusalem, Israel; 17 Genetics Institute, Emek Medical Center, Afula, Israel; 18 Rappaport Faculty of Medicine, Technion-Israel Institute of Technology, Haifa, Israel; 19 Sharett Institute of Oncology, Hadassah Hebrew University Hospital, Jerusalem, Israel; 20 Division of Gastroenterology, Rabin Medical Center-Beilinson Hospital, Petach Tikva, Israel; 21 Faculty of Medicine, Hebrew University, Jerusalem, Israel; 22 Susanne Levy Gertner Oncogenetics Unit, The Danek Gertner Institute of Human Genetics Chaim Sheba Medical Center, Tel-Hashomer, Israel

**Correction: Familial Cancer**

10.1007/s10689-024-00432-w

In the original version of this article, the given and family names of the author “Aasem Abu Shtaya” were incorrectly structured as “Shtaya, A.A” in the article citation but should have been ‘Abu Shtaya A’. The name was displayed correctly in all versions at the time of publication.

The original article has been corrected.

